# Application of conditional generative adversarial network to multi-step car-following modeling

**DOI:** 10.3389/fnbot.2023.1148892

**Published:** 2023-03-23

**Authors:** Lijing Ma, Shiru Qu

**Affiliations:** School of Automation, Northwestern Polytechnical University, Xi'an, China

**Keywords:** mixed traffic flow, unsupervised learning, deep learning, connected and autonomous vehicles, multi-step predictions, decision-making

## Abstract

Car-following modeling is essential in the longitudinal control for connected and autonomous vehicles (CAVs). Considering the advantage of the generative adversarial network (GAN) in capturing realistic data distribution, this paper applies conditional GAN (CGAN) to car-following modeling. The generator is elaborately designed with a sequence-to-sequence structure to reflect the decision-making process of human driving behavior. The proposed model is trained and tested based on the empirical dataset, and it is compared with a supervised learning model and a mathematical model. Numerical simulations are conducted to verify the model's performance, especially in the condition of mixed traffic flow. The comparison result shows that the CGAN model outperforms others in trajectory reproduction, indicating it can effectively imitate human driving behavior. The simulation results suggest that the introduction of CGAN-based CAVs improves the stability and efficiency of the mixed traffic flow.

## 1. Introduction

The development of connected and autonomous vehicles (CAVs) has become a topic of intense interest recently (SAE, 2021). CAVs have the potential to significantly improve the operation of transportation systems in various aspects, including traffic efficiency, safety, and sustainability. CAVs are equipped with advanced technologies such as sensors, cameras, and GPS systems, which allow them to collect and process vast amounts of data in real-time, enabling them to make informed decisions and respond quickly to changing road conditions. Such kind of new technologies (Wu D. et al., 2017; Yan et al., 2020; Du et al., 2021; Xu et al., 2021, 2022; Chen, 2022; Liu et al., 2022) has sprung up in recent years. One of the main advantages of CAVs is their ability to communicate with other vehicles and infrastructure, allowing them to operate more efficiently and safely. For example, CAVs can share real-time traffic information, such as traffic density, speed, and congestion, with other vehicles and traffic management systems, which can optimize traffic flow and reduce congestion.

The introduction of CAVs poses some significant challenges. One of the main challenges is that CAVs will operate in the mixed traffic environment, which includes both human-driven vehicles (HVs) and CAVs. This is because, at present, the majority of vehicles on the road are still human-driven. This creates a complex and dynamic traffic environment, where CAVs need to be able to interact and adapt to the driving behavior of HVs. To address this challenge, researchers have been developing data-driven models (Yang et al., 2019; Li et al., 2020) based on HVs' trajectory data to assist CAVs in adapting to the mixed traffic flow environment. These models are designed to imitate human driving behavior and perform realistic trajectory prediction, enabling CAVs to anticipate and respond to the driving behavior of HVs.

The outperforming data-driven car-following models are mainly established with prevailing machine learning approaches (Li et al., 2020), such as reinforcement learning and deep learning. In terms of reinforcement learning (RL) based models, the early studies go back to Wu C. et al. (2017) and Zhu et al. (2018). Then, inverse reinforcement learning (IRL) is introduced to car-following modeling (Gao et al., 2018; Huang et al., 2021), overcoming the difficulty in reward function designing. In terms of the deep learning approach, car-following models based on recurrent neural network (RNN) and its extended variants, such as long short-term memory (LSTM) and gated recurrent unit (GRU), embedding the memory effect of human driving behavior in car-following modeling (Wang et al., 2017, 2019; Zhou et al., 2017; Huang et al., 2018), have been demonstrated to be superior to the models based on conventional neural networks. Recently, to improve the decision-making process, some studies have proposed car-following models with advanced structures, for example, encoder-decoder (Gu et al., 2020; Ma and Qu, 2020), attention mechanism (Lin et al., 2021; Shi K. et al., 2022), and transformer (Sachdeva et al., 2022; Zhu et al., 2022). However, in the supervised learning framework, the empirical data only contains limited observations, lacking abnormal states, such as almost rear-end collision. As a result, in the testing process, the data distribution can be very different from the training data, and the learned model has no clue what to do when a slight deviation occurs. Moreover, there can be mistakes or poor behaviors in the empirical data, such as unnecessarily slamming on the brakes, and the learned model may copy these errors.

Generative adversarial networks (GAN) (Goodfellow et al., 2020) and variants achieve significant success in computer vision, inspiring researchers to explore their application in other fields: dialogue generation (Li et al., 2017), stock price prediction (Fu et al., 2019), trajectory generation (Gao et al., 2022), etc. There are two components in the framework of GAN: the generator network and the discriminator network. As the generator learns to generate data distribution similar to the training data, the discriminator tries to distinguish the generated data from the real one. They update adversarially in an unsupervised manner, potentially improving the deficiencies of supervised learning. Currently, in the field of longitudinal control for CAVs, car-following models based on GANs (Kuefler et al., 2017; Greveling, 2018; Zhou et al., 2020; Bhattacharyya et al., 2022; Mo and Di, 2022; Shi H. et al., 2022) are state-of-the-art. Nevertheless, few studies ponder over the continuity of time series. In other words, the discriminator needs to not only estimate whether the generated data is like human driving behavior but also judge whether it is the correct action. Moreover, the generator is better to be meticulously designed considering the decision-making process of car-following behavior.

Hence, we outline the potential limitations: (i) The discriminator of the previous GAN-based model cannot evaluate whether the generated action is appropriate for a given situation. (ii) The ordinary design of the generator cannot cover the decision-making process of car-following behavior. (iii) The car-following models are constructed for CAVs but seldom verified in the mixed traffic flow environment.

The core task of our study is building an outperforming car-following strategy for CAVs in mixed traffic flow. We propose a multi-step car-following model based on conditional GAN to bridge the above gaps. The main contributions are: (i) A variant of GAN, conditional GAN (CGAN), is applied to car-following modeling to better estimate the generated action due to its improved discriminator structure. (ii) The Encoder-Decoder framework is introduced to design the generator network, which imitates the decision-making process of car-following behavior and gives multi-step predictions. (iii) The platoon simulation and a periodic boundary condition are introduced for numerical simulation, and the performance of the proposed CGAN model is verified, demonstrating its availability for CAVs in mixed traffic flow.

The rest of this paper is organized as follows. Section 2 is dedicated to reviewing related work. Section 3 proposes the architecture and configuration of the CGAN car-following model. In Section 4, the proposed model is trained with the empirical dataset, and the models are compared in terms of prediction accuracy. Section 5 presents the performance of the model based on numerical simulation. In Section 6, our findings are concluded.

## 2. Related work

In recent years, the research on longitudinal control of CAVs has gradually changed from establishing various mathematical models (Brackstone and McDonald, 1999; Saifuzzaman and Zheng, 2014) to focusing more on building data-driven models based on HVs' trajectory data (Yang et al., 2019; Li et al., 2020). Data-driven car-following models do not require mathematical formulas or calibration but instead can extract behavior from mass field data. Machine learning approaches, including nonparametric regression, support vector regression (SVR), reinforcement learning (RL), and deep learning (DL), have become popular for car-following behavior modeling due to their success in outperforming mathematical models.

Nonparametric regression-based models. The main concept behind nonparametric regression car-following models is to predict vehicle positions as a way of reproducing traffic dynamics. The estimation of vehicle trajectories using locally weighted regression was initially presented by Toledo et al. (2007) and later enhanced by Papathanasopoulou and Antoniou (2015). A nonparametric car-following model based on k-nearest neighbor was introduced and experimentally validated using field data by He et al. (2015).

Support vector regression-based models. A universal learning method called Support Vector Machine (SVM) is based on statistical learning theory, and its application for regression problems is SVR. In the case of car-following modeling, Wei and Liu (2013) introduced SVR to investigate the asymmetric characteristic in car-following behavior since it can discover inherent relationships among the variables in a dataset. The SVR car-following model uses space headway, the follower's speed, and relative speed as inputs and produces the follower's speed as output.

Reinforcement learning-based models. Wu C. et al. (2017) presented Flow, a computational framework that simplifies the development of controllers for autonomous vehicles in complex traffic scenarios with deep reinforcement learning. Flow integrates the micro traffic simulator SUMO and the deep RL library rllab, enabling the efficient design of traffic tasks and the creation of controllers for mixed-autonomy traffic scenarios. Zhu et al. (2018) proposed an autonomous car-following framework based on deep reinforcement learning that trains an RL agent using historical driving data to learn a car-following model. The model can map speed, relative speed, and inter-vehicle spacing to the acceleration of a following vehicle and outperforms traditional and recent data-driven car-following models, reproducing human-like behavior with higher accuracy and adaptability. The study highlights the potential of reinforcement learning to improve autonomous driving algorithms and traffic-flow models. Gao et al. (2018) discussed the challenge of designing a car-following decision-making system for complex traffic conditions in the field of autonomous driving. It presents a method based on an inverse reinforcement learning algorithm to establish driver data reward functions, analyzes driving characteristics and following strategies, and demonstrates the method's effectiveness through simulation in a highway environment. Huang et al. (2021) proposed an internal reward function-based driving model with inverse reinforcement learning to learn personalized reward functions for individual human drivers from driving data. A structural assumption about human driving behavior is proposed, which focuses on discrete latent driving intentions to make maximum entropy IRL tractable. The proposed method outperforms general modeling and baseline methods and demonstrates the importance of considering interactive behaviors among the ego and surrounding vehicles in estimating generated trajectories.

Deep learning-based models. Zhou et al. (2017) proposed a recurrent neural network-based model for predicting traffic oscillation in car-following. The model outperforms classical car-following models under different driver characteristics, demonstrating the efficacy of data-driven neural network approaches for traffic flow dynamics. Huang et al. (2018) proposed an LSTM-based car-following model considering asymmetric driving behavior. The model is calibrated and validated with a real dataset and outperforms other models in reproducing realistic traffic flow features. Ma and Qu (2020) proposed a car-following model using sequence-to-sequence (seq2seq) learning. The model includes spatial anticipation and improves platoon simulation accuracy and traffic flow stability, making it an effective solution for simulating car-following behavior and suitable for traffic analysis and simulation. Zhu et al. (2022) proposed a long-sequence car-following trajectory prediction model based on an attention-based Transformer model, which outperforms traditional models in terms of accuracy and can be used to simulate traffic flow and develop intelligent vehicles.

In brief, machine learning approaches, particularly reinforcement learning and deep learning, have successfully outperformed mathematical models in recent studies and show promise in improving autonomous driving algorithms and traffic flow models.

## 3. Car-following model based on CGAN

### 3.1. Architecture

In general, Conditional Generative Adversarial Network (CGAN) (Mirza and Osindero, 2014) is a type of neural network used in deep learning to generate new data samples similar to a given training set. CGANs are an extension of Generative Adversarial Networks (GANs) and are conditioned on additional input variables, such as class labels, images, or text. The main components of a CGAN are the generator and the discriminator. The generator and discriminator are trained in a competitive process. The generator tries to produce samples similar to the real data while also satisfying the given conditions. The discriminator tries to correctly classify whether the samples are real or fake while also taking the conditional variables into account. By incorporating additional information into the generation process, CGANs are able to produce more realistic and diverse outputs that are tailored to specific conditions. CGANs are used in various tasks, such as image-to-image translation, text-to-image synthesis, and super-resolution.

Compared to supervised learning, which is a common approach for car-following models, CGAN has the advantage of being able to learn from unlabeled data and can potentially generate more diverse and realistic driving behaviors. Compared to a traditional GAN, the advantage of CGAN is that it can generate more specific and tailored outputs based on the additional conditioning variables, making it a more precise and effective model. Additionally, CGAN can better handle situations where the data has a complex structure, and additional context is required for the generation task. However, the weakness of CGAN is that it can be more complex and challenging to implement than the above intelligent networks, as it may require more computational resources to train. Overall, CGAN is a promising approach for car-following models in autonomous driving.

**Figure 1:**
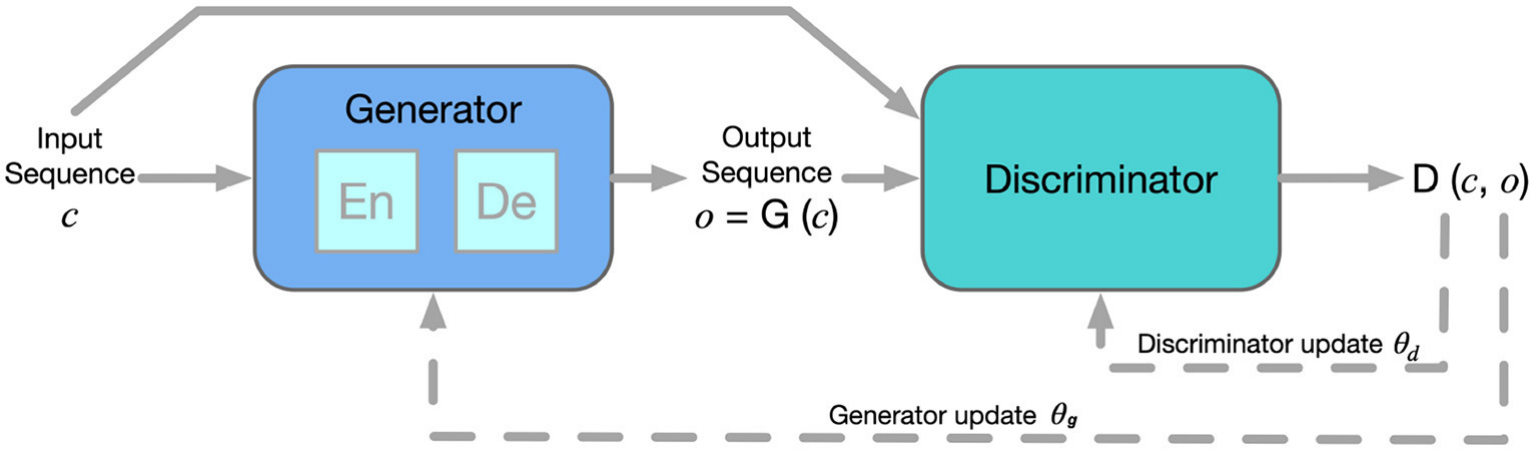
CGAN architecture for car-following modeling.

In our study, we improve the Generator to an Encoder-Decoder framework, which predicts the action (output sequence *o*) with a given state (input sequence *c*). Also, the input sequence is additional information for the Discriminator in the CGAN structure. The Discriminator learns to distinguish the output sequence from the real sample (i.e., ensuring the action is similar to human behavior) and estimate whether it corresponds to the input sequence (i.e., ensuring the action is correct). The overall framework for CGAN-based car-following modeling are presented in Figure 1.

In this way, for car-following modeling, the Discriminator updates to have a better judgment of the generated behavior, and the Generator obtains feedback from the Discriminator and updates to imitate human driving behavior better. Therefore, the training process of CGAN is a min-max game, as the objective functions given in Equations (1)–(3).


(1) $$\begin{array}{l} D^{*}=\arg\underset{D}{\max}V\left(G,D\right) \end{array}$$

(2) $$\begin{array}{l} G^{*}=\arg\underset{G}{\min}\underset{D}{\max}V\left(G,D\right) \end{array}$$

(3) $$\begin{array}{l} V\left(G,D\right)={\text{E}}_{\left(c,o\right)\sim P_{data}}\left[\log D\left(c,o\right)\right]+{\text{E}}_{c\sim P_{data},x\sim P_{G}}\left[\log\left(1-D\left(c,o\right)\right)\right] \end{array}$$

Where *V*(*G, D*) is the loss function, denoting the difference between ground truth and generated data. *P*_*data*_ is the real data distribution, and *P*_*G*_ is a distribution learned by the Generator. *D*(*c, o*) represents the probability that *o* is from the real data.

Based on the architecture of CGAN, the detailed training process is presented in Algorithm 1. θ_*g*_ and θ_*d*_ represent parameters in the Generator and the Discriminator, which are updated in training iterations.

**Algorithm 1:**
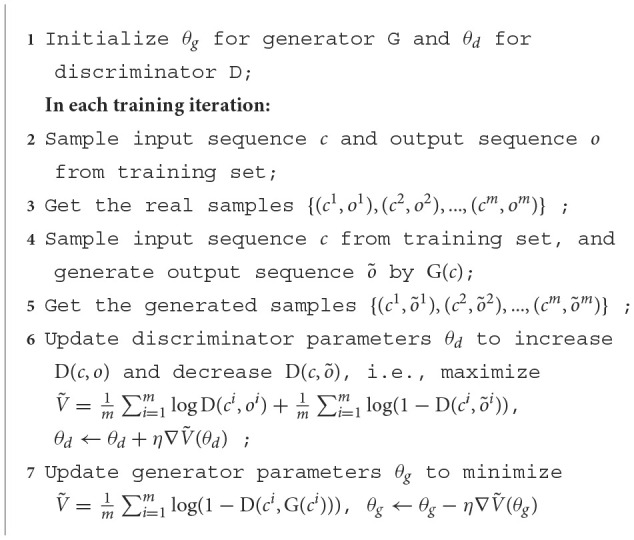
CGAN training.

### 3.2. Configuration

**Figure 2:**
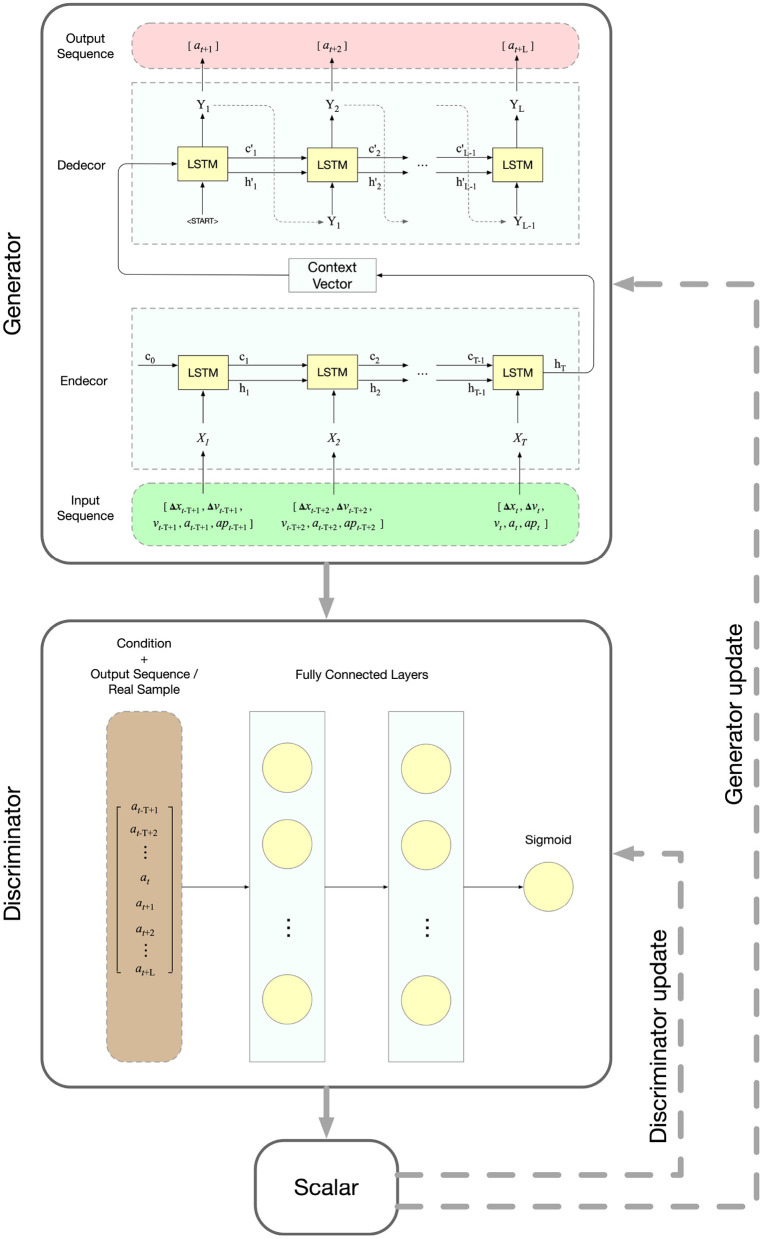
The framework of CGAN car-following model.

Figure 2 shows the detailed framework of CGAN car-following model. The structure of the Generator is a sequence-to-sequence learning (Seq2Seq) model, which takes multi-step driving states as input and outputs multi-step actions. For clarity, the basic structure of the Seq2Seq model and long short-term memory (LSTM) unit can be found in our previous study (Ma and Qu, 2020).

Ma and Qu (2020) has investigated the configuration of input and output variables based on literature review and the car-following process, determining the distance and speed-related variables as the input and the acceleration as the output. For CAVs, the technology enables the vehicle to detect acceleration besides distance and speed. Therefore, the gap distance (Δ*x*), relative speed (Δ*v*), speed (*v*), acceleration (*a*), and acceleration of leading vehicle (*ap*) are adopted as the input variables, and the acceleration (*a*) is adopted as the output variable. Furthermore, according to the decision-making process studied in Ma and Qu (2020), the input and output of the Generator are sequences, and the lengths are T and L, respectively. The mapping function is formulated in Equation (4):


(4) $$\begin{array}{l} a_{t+1},...,a_{t+\text{L}}=f(\;\Delta x_{t-\text{T}+1},...,\Delta x_{t},\\ \;\Delta v_{t-\text{T}+1},...,\Delta v_{t},\\ \;v_{t-\text{T}+1},...,v_{t},\\ \;a_{t-\text{T}+1},...,a_{t},\\ \;ap_{t-\text{T}+1},...,ap_{t}\;) \end{array}$$

The structure of the Discriminator is a fully connected neural network with two hidden layers. The output layer is a sigmoid function, giving the scalar that indicates the probability of the action belonging to real or generated data. In terms of the input of the Discriminator, part of the input sequence of the Generator (i.e., the action at previous time steps), which is the condition, is concentrated with the Generator's output sequence (i.e., the predicted action for future time steps) and forms the input. The mapping function is formulated in Equation (5):


(5) $$\begin{array}{l} D=g\left(a_{t-\text{T}+1},...,a_{t},a_{t+1},...,a_{t+\text{L}}\right) \end{array}$$

## 4. Case study

### 4.1. Data preparation

Data-driven car-following models for CAVs need to learn from the real human-driven vehicle trajectory data. The Next Generation Simulation (NGSIM) (FHWA, 2008) dataset is a commonly used empirical dataset, a detailed and high-fidelity traffic dataset collected from the real world. One of the primary benefits of using NGSIM for data-driven car-following modeling is that it provides a realistic and comprehensive view of traffic dynamics, which is critical for developing accurate car-following models for CAVs. This allows car-following models to be validated and tested under a wide range of scenarios and conditions, which can help improve the models' robustness and reliability. Another advantage of using NGSIM is that it provides a standardized benchmark dataset for researchers to compare and evaluate their car-following models. This allows researchers to assess the accuracy and effectiveness of their models and compare them to other existing models in a fair and standardized manner.

The Interstate 80 (I-80) Highway dataset is one of the several datasets collected under the NGSIM program. It was collected on April 13, 2005, eastbound I-80 in the San Francisco Bay Area in Emeryville, California. The study area is approximately 400 m in length.

Montanino and Punzo (2015) reconstructed the trajectory of the NGSIM dataset to repair the measurement errors in the original dataset and ensure that the trajectory data conforms to vehicle kinematics and microscopic traffic dynamics. We take this reconstructed I-80 dataset (Montanino and Punzo, 2013), which contains 15 min of vehicle trajectory data (4:00 p.m. to 4:15 p.m.) at a data resolution of 10 Hz. Since our study only focuses on longitudinal control, we extract car-following events from the dataset with the following four steps:

- Trajectories on five regular lanes (from Lane 2 to Lane 6) are picked out, getting rid of the trajectories on Lane 1, as it is a High Occupancy Vehicle (HOV) lane, and the traffic may be irregular.
- Vehicle lengths no more than 5 m are kept, excluding trucks.
- Clearance distances of more than 120 m are filtered out, avoiding unimpeded traffic situations.
- Continuous car-following times of fewer than 30 s are filtered out, eliminating the influence of lane-changing.

**Figure 3:**
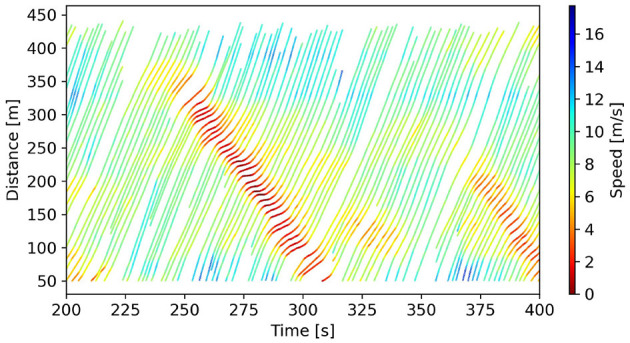
The spatio-temporal map of some car-following events from Lane 2.

As a result, we obtain 1,386 car-following events involving 662,378 trajectory data points. Each data point contains trajectory information at that time step. Figure 3 shows the spatio-temporal map of some car-following events. These 1,386 car-following events need to be split into two parts to train and test data-driven models. Due to the required independent testing procedure, trajectories from one lane can be selected to compose the test dataset. Therefore, 332 car-following events collected from Lane 2 are chosen as the testing dataset, and the trajectory samples from the remaining lanes (from Lane 3 to Lane 6) form the training dataset, with a total of 1,054 car-following events.

### 4.2. Model training

The CGAN car-following model is trained based on the procedure in Algorithm 1, implemented with Python. With experimental optimization, the hyperparameters are tuned and listed as follows:

- Optimizer. The adaptive optimizer Adam (Kingma and Ba, 2014) is utilized, as it is effective in training car-following models (Ma and Qu, 2020; Shi H. et al., 2022). In this study, the parameters in Adam are tuned as *lr* = 0.00005, *beta*_1_ = 0.9, *beta*_2_ = 0.999, *epsilon* = 1*e*−08, *decay* = 0.0.
- Neuron. In the Generator, the number of neurons for the LSTM units in the Encoder-Decoder framework is 32. In the Discriminator, the number of neurons for the hidden layers is 64.
- Activation function. In the Generator, the hyperbolic tangent function *tanh*(·) is chosen as the activation function. In the Discriminator, two hidden layers adopt the leaky ReLU function, and the output layer adopts the sigmoid function.
- Epoch. At each epoch, the learning algorithm traverses the entire training dataset. The number of epochs is 2000, and the batch size is 128. As the best convergence and balance of the Generator and the Discriminator is fleeting, at the end of each epoch, the performance of the Generator is presented to capture the best parameters.

The Mean Squared Error (MSE) is a commonly used metric for trajectory prediction tasks. It is suitable to evaluate the performance of the Generator. To directly measure the accuracy of the predicted trajectory, we formulate the MSE based on the spatio-temporal data, as shown in Equation (6), where the average errors between the actual and predicted locations are calculated. The predicted location is constructed with the discrete-time kinematic rule based on the generated action. The detail is presented in Equation (7).


(6) $$\begin{array}{l} MSE=\frac{1}{M}\overset{M}{\underset{t=1}{\sum }}{\left[x_{t}-{\hat{x}}_{t}\right]}^{2} \end{array}$$

(7) $$\{\begin{array}{l} {\hat{v}}_{t+1}={\hat{v}}_{t}+{\hat{a}}_{t+1}\Delta t\\ {\hat{x}}_{t+1}={\hat{x}}_{t}+{\hat{v}}_{t}\Delta t+\frac{1}{2}{\hat{a}}_{t+1}\Delta t^{2} \end{array}$$

Where â is the generated action, i.e., acceleration. $\hat{v}$ and $\hat{x}$ denote the predicted speed and location. *x*_*n*_ represents the observed location.

In the training process, MSE is taken as the evaluation indicator for configuration optimization. Based on the ranges of historical time steps and prediction horizons investigated by Ma and Qu (2020), we test different lengths of input and output sequences for the Generator. It turns out that T = 50 and L = 10 achieve considerable performance.

### 4.3. Models comparison

To verify the performance of the CGAN car-following model, we applied it to trajectory reproduction based on the test dataset. The experiment is also conducted on other car-following models, including the Seq2Seq model and a classic mathematical model, and their performances are compared.

The intelligent driver model (IDM) is a prevailing mathematical car-following model first proposed by Treiber et al. (2000), as formulated in Equations (8)–(9). The predicted acceleration (*a*) is calculated with the observed gap distance (Δ*x*), relative speed (Δ*v*), and speed (*v*). The parameters include maximum acceleration (ã), comfortable deceleration ($\tilde{b}$), desired speed (ṽ), desired time headway (*t*_0_), and safe gap distance (*s*_0_), which can be calibrated with the training dataset.


(8) $$\begin{array}{l} a=ã\left[1-{\left(\frac{v}{ṽ}\right)}^{4}-{\left(\frac{S\left(v,\Delta v\right)}{\Delta x}\right)}^{2}\right] \end{array}$$

(9) $$\begin{array}{l} S\left(v,\Delta v\right)=s_{0}+t_{0}v-\frac{v\Delta v}{2\sqrt{ã\tilde{b}}} \end{array}$$

IDM is a classic car-following model widely used as a comparative model in recent studies (Zhou et al., 2017; Zhu et al., 2018, 2022; Ma and Qu, 2020). IDM captures some of the key behaviors exhibited by human drivers, such as maintaining a safe distance from the vehicle in front, adjusting speed to avoid collisions, and accelerating and decelerating in response to changes in traffic flow. This makes it a useful baseline for evaluating the performance of more complex and advanced car-following models. In this study, we calibrate the parameters of IDM with genetic algorithm (GA) (Mitchell, 1998), and the values are given in Table 1.

**Table 1:** Calibrated parameters of the IDM.

**IDM**	**ã**	** $\tilde{b}$ **	**ṽ**	***t*_0_**	***s*_0_**
Calibrated value	2.02	1.43	22.89	1.40	2.75

**Figure 4:**
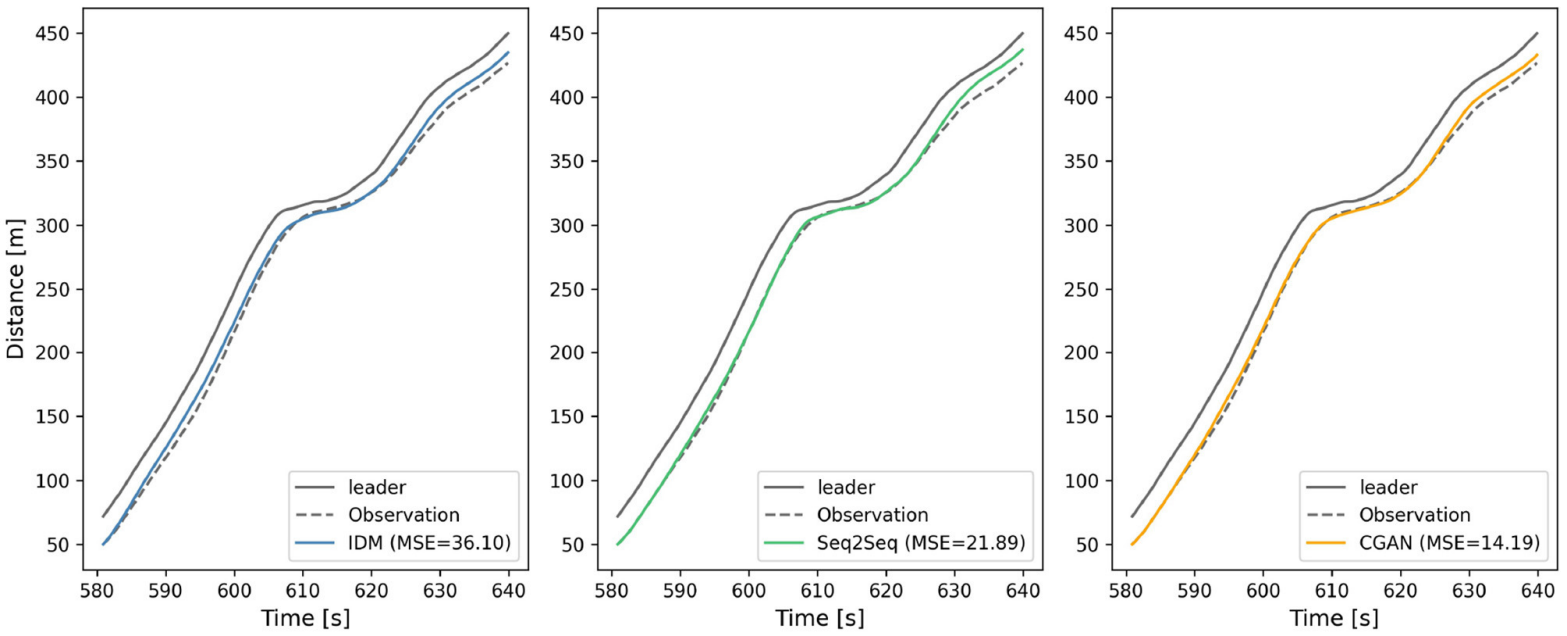
Spatio-temporal diagrams (Vehicle 1898).

**Figure 5:**
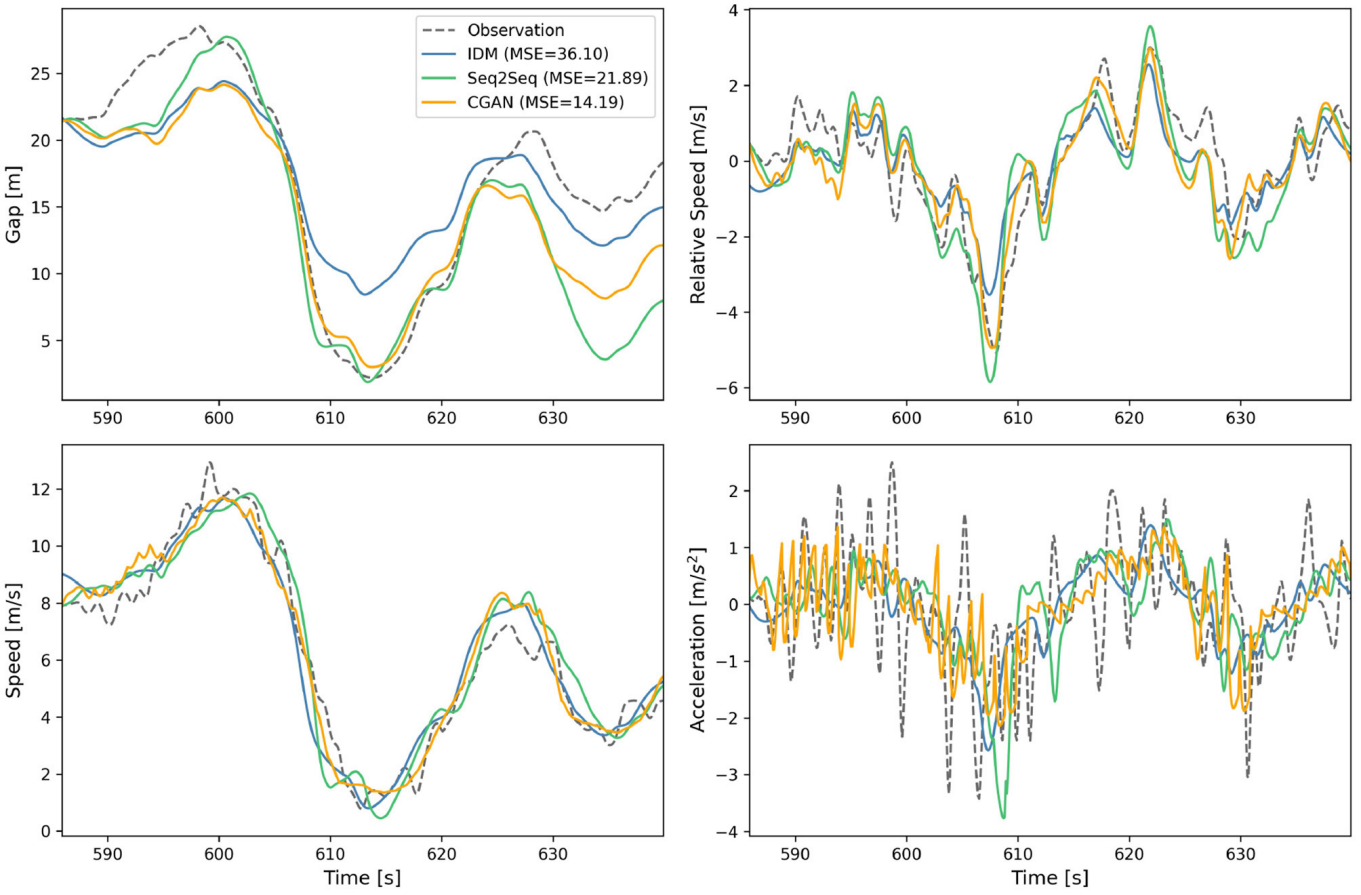
Trajectory profiles of Vehicle 1898.

The models' performances are evaluated by the indicator MSE. To visually illustrate the performance, we randomly select a car-following event (the subject vehicle is Vehicle 1898) and plot the spatio-temporal diagrams in Figure 4. The reproducing trajectories with IDM, Seq2Seq, and CGAN are compared with the observation, and the MSE are 36.10, 21.89, and 14.19, respectively. Figure 5 presents the trajectory profiles, showing the deviations at speed and acceleration levels besides the distance level. It reveals that all three models imitate the primary behavior well, while the trajectory reproduced by CGAN is more in line with the observation.

In order to draw a general comparison, we conduct statistical analysis on the MSE values of the test dataset (332 car-following events), as presented in Table 2. For IDM, Seq2Seq, and CGAN, the mean MSE values are 26.74, 21.60, and 19.58, respectively. The standard deviation and the percentiles show that the MSE values of CGAN are more densely distributed than the values of the other two models.

**Table 2:** Statistics results of the performance.

**Model**	**Mean (SD)**	**Min**	**Max**	**Percentile [25%, 50%, 75%]**
IDM	26.74 (38.70)	1.21	374.28	[8.28, 14.87, 29.91]
Seq2Seq	21.60 (28.42)	0.73	233.29	[7.57, 12.27, 25.23]
CGAN	19.58 (22.73)	0.33	229.12	[6.66, 13.01, 24.18]

It is widely acknowledged that IDM is an outstanding mathematical model that provides reliable car-following performance. Moreover, it has been proved that Seq2Seq achieves outperformance for multi-step prediction considering the decision-making process of human driving behavior. Therefore, the comparison indicates that the proposed CGAN car-following model is available as it yields an excellent fitting quality with the empirical driving data.

The case study results show that the proposed multi-step car-following model based on CGAN outperforms existing car-following models (IDM and Seq2Seq) in terms of MSE for the test dataset. The proposed model also produces trajectory profiles that are more in line with the observation, indicating that it provides more accurate and effective car-following strategies for CAVs. Moreover, the proposed CGAN-based model has a more flexible and diverse generation of car-following strategies, depending on the specific traffic flow conditions and requirements. This means that the proposed model can adapt to the dynamic and complex nature of mixed traffic flow, further improving the safety and efficiency of CAVs on the road.

## 5. Simulations

### 5.1. Platoon simulation

Platoon simulation is a vital strategy for car-following model testing, and its implementation differs from the operation of vehicle pairs' trajectory reproduction. In trajectory reproduction, the predicted action is based on the actual states of the preceding vehicle. Nevertheless, in platoon simulation, except for the first following vehicle, the other following vehicles are simulated based on their initial states and the simulation results of their preceding vehicles. In this way, the errors induced by the controlling model may be exaggerated to test the model's performance. Therefore, to further explore the performance of the proposed car-following model, we conduct platoon simulation assuming the following vehicles are controlled by the CGAN.

The simulation design is similar to the study in Treiber et al. (2006), where the platoon includes 100 vehicles, and the first vehicle is an externally controlled leader. All the vehicles' lengths are set to be 5 m. The simulation lasts 2000 s, and the update interval is 0.1 s same as the resolution of the empirical dataset. The first vehicle travels at a constant speed *v* = 15.3 m/s for the first 50 s, then it decelerates to *v* = 14.0 m/s with *a* = −0.65 m/s^2^ and continues to move at this speed until the simulation ends. The initial states of other vehicles are assumed to be equilibrium at speed *v*^*e*^ = 15.3 m/s. As in previous studies (Treiber et al., 2006; Ma et al., 2023), the corresponding equilibrium gap distance (Δ*x*^*e*^) is derived from the IDM. As the relative speed and acceleration are both zeros at the equilibrium state, they are substituted into Equations (8), (9), obtaining Δ*x*^*e*^ as in Equation (10). Calculated with the calibrated parameters in Table 1, all the initial gap distances between adjacent vehicles are 27.02 m.


(10) $$\begin{array}{l} \Delta x^{e}=\frac{s_{0}+v^{e}t_{0}}{\sqrt{1-{\left(\frac{v^{e}}{ṽ}\right)}^{4}}}, \end{array}$$

**Figure 6:**
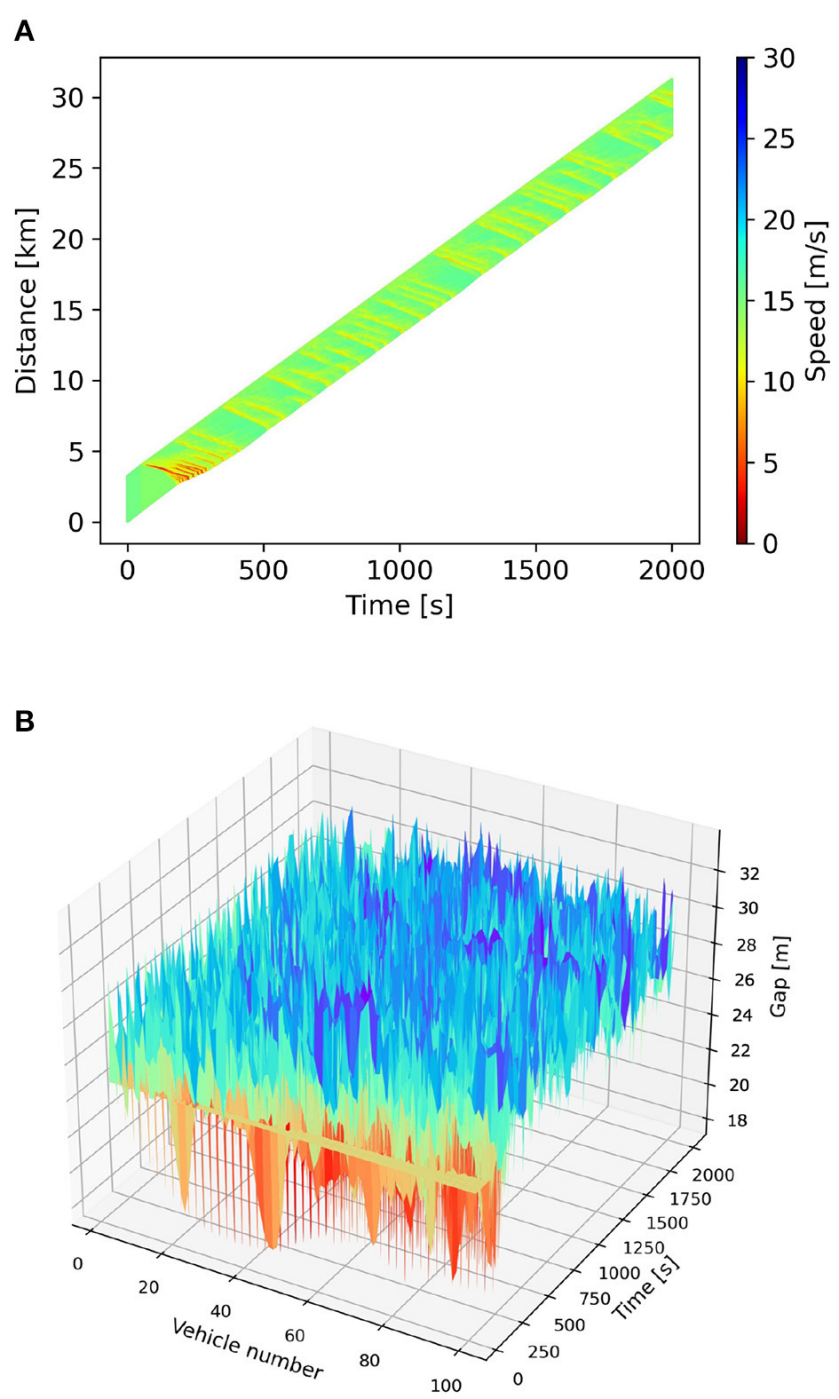
Platoon simulation result. **(A)** Space-time diagram. **(B)** Space-time evolution of gap distance.

The platoon simulation is conducted with the CGAN model, and the result is summarized in Figure 6. Figure 6A visualizes the simulated trajectory set as a space-time diagram, and Figure 6B presents the space-time evolution of gap distance. It shows that, influenced by the deceleration of the first vehicle, the following vehicles in the platoon experience traffic oscillation over a period of time. This is a typical phenomenon as the disturbance propagates upstream. Soon afterward, trajectories are smoothed with less oscillation, and the speed and gap fluctuations become weaker with time.

**Figure 7:**
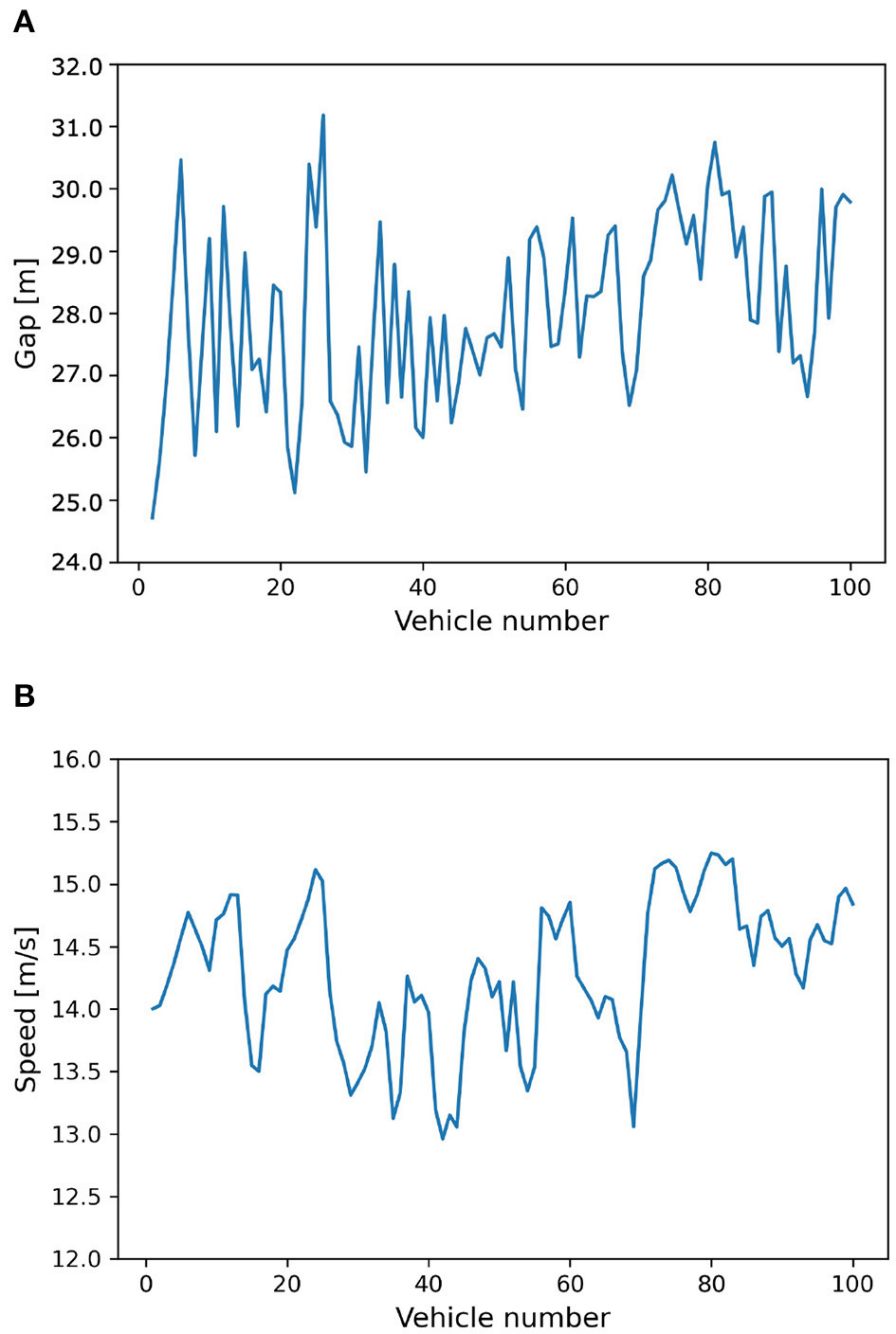
Snapshot profiles at 2,000 s. **(A)** Snapshot of gap distance. **(B)** Snapshot of speed.

Besides, to clearly observe the final states, we plot the snapshots of gap distance and speed of all vehicles at 2000 s, i.e., the end of the simulation, as presented in Figure 7. It shows that the simulated vehicles are not running in a duplicate way, and their gap distances and speeds vary but stay in specific ranges. The platoon simulation result indicates that the proposed CGAN model is available as it can reproduce the actual traffic flow. Nevertheless, the simulated speeds do not converge to a constant, and the model's stability is further verified in the next section.

The results of the platoon simulation indicate that the CGAN model is capable of reproducing the traffic oscillation and smoothening phenomena that occur in a platoon when the first vehicle decelerates. This shows that the proposed model can accurately capture the dynamics of traffic flow and generate car-following strategies that adapt to the complex nature of traffic flow, improving transportation safety and efficiency.

### 5.2. Simulation in a mixed traffic flow environment

As CGAN car-following model is constructed for CAVs, it should be tested in a mixed traffic flow environment, including HVs and CAVs, to verify its performance. To conduct mixed traffic flow simulation, Ma et al. (2023) has designed a periodic boundary condition. It is an essential experimental approach for traffic flow stability analysis (Li and Shrivastava, 2002), which is consistent with the assumption of an infinitely long platoon in the theoretical stability analysis. This study uses the same periodic boundary condition to verify the CGAN model.

**Figure 8:**
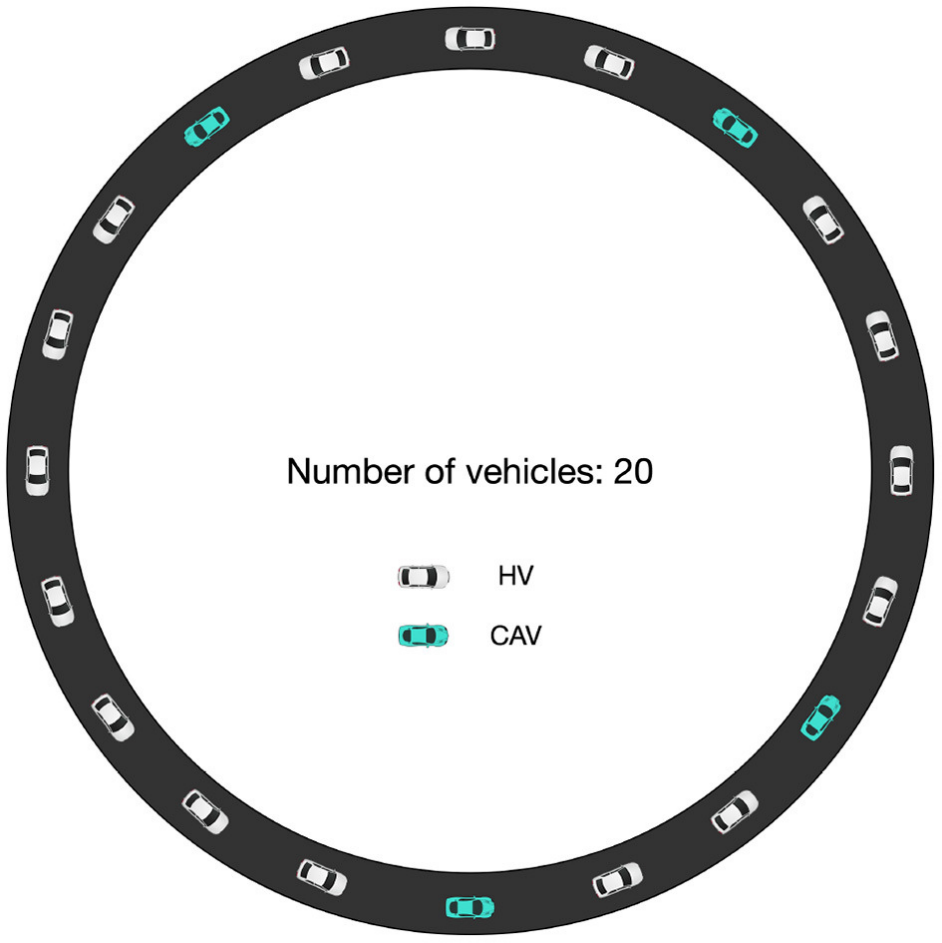
Schematic of the periodic boundary condition for mixed traffic flow (for example, the penetration rate of CAVs is 20%).

A circular road without ramps is used, and 20 vehicles operate on the road with a head-to-tail structure. The assumption of vehicle length and the initial speed and gap distance is the same as in the platoon simulation: *l* = 5 m, *v*^2^ = 15.3 m/s, and Δ*x*^*e*^ = 27.02 m. Thus, the length of the circular road is 640.4 m. At 50 s, one vehicle is randomly chosen, and a disturbance is applied, letting it decelerates with 0.65 m/s^2^ until its speed equals 14.0 m/s. Then, all the vehicles on the road follow the vehicle in front according to its car-following strategy until the end. Among the vehicles, HVs and CAVs are randomly distributed. The HVs are controlled by Seq2Seq car-following model, as this model is proven to be outstanding in imitating human driving behavior. The penetration rate for CAVs varies from 0% to 100%, i.e., *p* = 0, 0.2, 0.4, 0.6, 0.8, 1, representing different structures of mixed traffic flows. The schematic is presented in Figure 8, taking the penetration rate of CAVs being 20% as an example.

**Figure 9:**
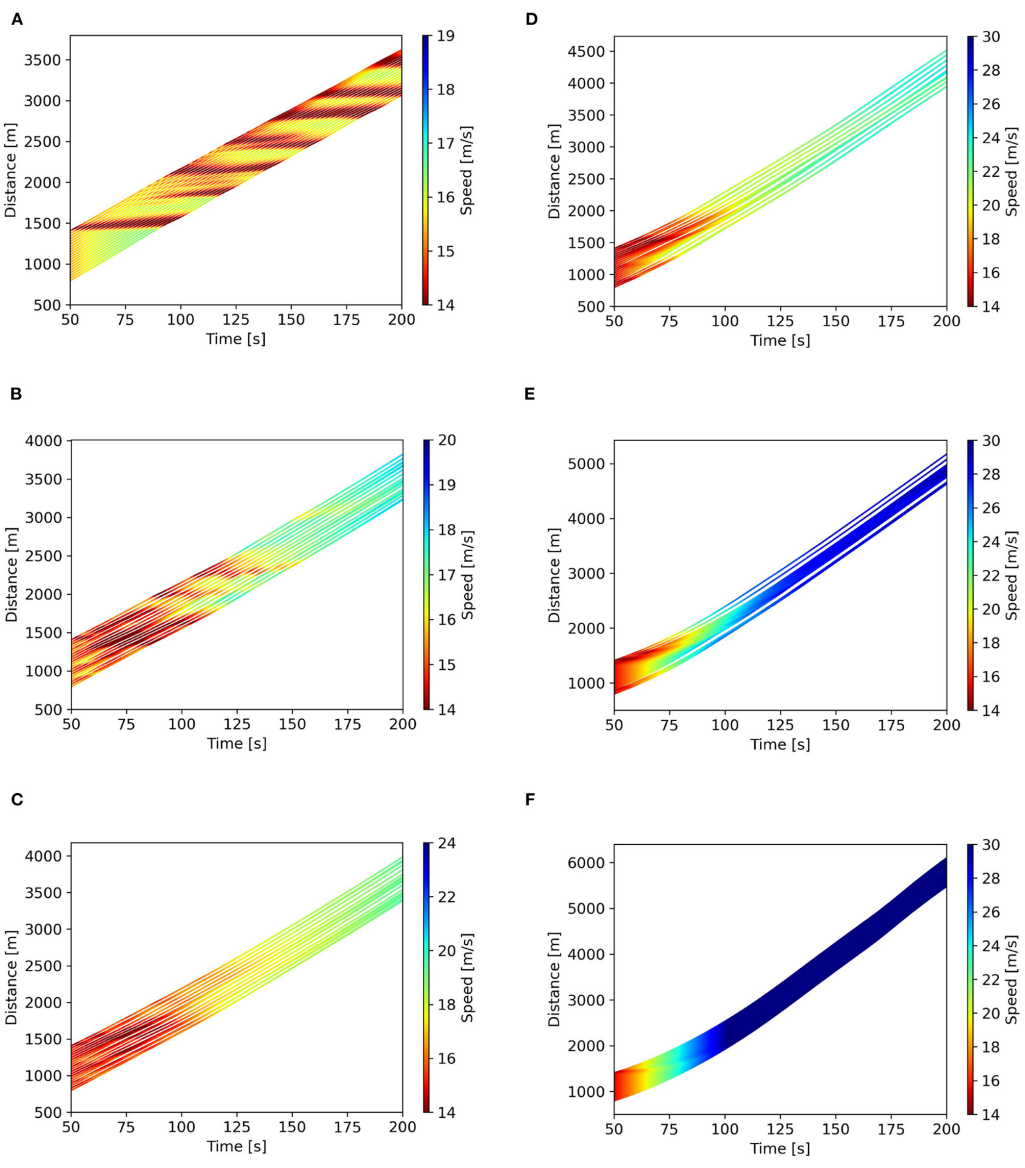
Space-time diagrams for different penetration rates of CAVs. **(A)** *p* = 0%, **(B)** *p* = 20%, **(C)** *p* = 40%, **(D)** *p* = 60%, **(E)** *p* = 80%, and **(F)** *p* = 100%.

The simulated trajectories of different penetration rates of CAVs are obtained, as presented in the space-time diagrams in Figure 9. For clarity, the initial states of the first 50 s are not necessary to be shown. The simulation results show that the average running speed of vehicles increases when the penetration rate of CAVs rises. It can be found in Figure 9A that the platoon with 100% HVs experiences oscillations (stop-an-go waves) caused by the perturbation. The introduction of CAVs smooths the trajectories, as shown in Figures 9B–E. With regard to 100% CAVs in Figure 9F, it reaches the most mileage than other structures, which means the traffic flow becomes more efficient.

In terms of the stability of CAVs, it is hard to have the CGAN model be analytically solved, because of its black-box nature. But in fact, this periodic boundary simulation environment has been determined to be a feasible method to validate the stability of the car-following model (Ma et al., 2023). It can be seen from Figure 9F that the effect of the perturbation is short-lived when all vehicles are controlled by the CGAN model, which indicates the stability of the CGAN model. As a result, the stability of mixed traffic flow can be improved with the introduction of CGAN-based CAVs.

**Figure 10:**
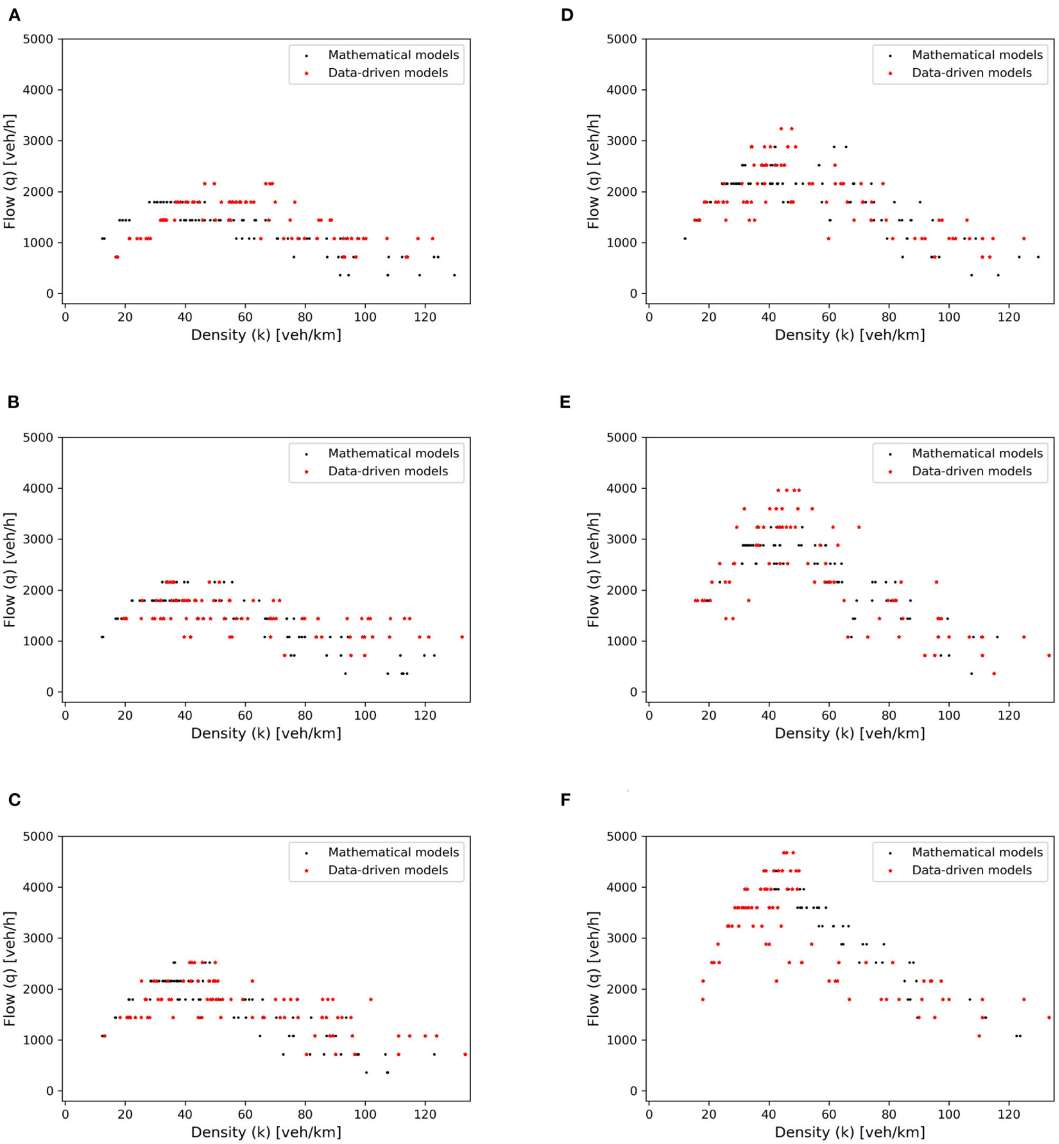
Fundamental diagrams for different penetration rates of CAVs. **(A)** *p* = 0%, **(B)** *p* = 20%, **(C)** *p* = 40%, **(D)** *p* = 60%, **(E)** *p* = 80%, and **(F)** *p* = 100%.

To explore the capacity of mixed traffic flow, we set two virtual detectors on the circular road with an interval of 100 m between them. During traffic simulation, a detector computes flow-density values at that location. Consequently, the scatter points for the fundamental diagram can be drawn, as the red points presented in Figure 10. The fundamental diagrams derived from this simulation are compared with the diagrams simulated with mathematical car-following models, i.e., the black scatters in the figures. For simplicity, the contrasted group is not presented here in detail since it is depicted in Ma et al. (2023). The two groups show consistency, indicating the reasonability of proposed data-driven models in terms of traffic flow simulation. In addition, with the increase of CAVs' proportions, the maximum capacity shown in the fundamental diagrams gradually rises. Therefore, the performance of the CGAN model is verified with the efficiency improvement of mixed traffic flow.

## 6. Conclusions

This study focuses on exploring the application of CGAN to multi-step car-following modeling. Based on the advantages of CGAN architecture and Seq2Seq structure, the proposed model aims to become a potential framework of longitudinal control for CAVs. As the mixed traffic flow of HVs and CAVs is an inevitable traffic environment nowadays, we check out the CAVs' performance in such circumstances. The main findings can be concluded as follows:

- The case study shows that the CGAN model outperforms the mathematical models in trajectory reproduction. This indicates that it can effectively imitate human driving behavior.
- The CGAN model overcomes the deficiency of supervised learning-based models in car-following modeling. The comparison of models suggests the CGAN model is a promising approach to substitute supervised learning models in trajectory prediction.
- Platoon simulation shows that the CGAN model reproduces the oscillation and smoothing of traffic flow. It suggests that the proposed model is practicable in natural traffic scenes.
- The performance of the CGAN model is further verified in the mixed traffic flow environment, conducted in a periodic boundary condition. The results suggest that the introduction of CGAN-based CAVs improves the stability and efficiency of the mixed traffic flow.

## Data availability statement

Publicly available datasets were analyzed in this study. This data can be found at: https://data.transportation.gov/stories/s/i5zb-xe34; http://www.multitude-project.eu/exchange/101.html.

## Author contributions

LM: conceptualization, methodology, software, formal analysis, investigation, resources, data curation, writing—original draft, and visualization. SQ: conceptualization, methodology, validation, writing—review and editing, supervision, and project administration. All authors contributed to the article and approved the submitted version.

## References

[B1] Bhattacharyya R. Wulfe B. Phillips D. J. Kuefler A. Morton J. Senanayake R.. (2022). Modeling human driving behavior through generative adversarial imitation learning. IEEE Trans. Intell. Transport. Syst. 24, 2874–2887. 10.1109/TITS.2022.322773832899773

[B2] Brackstone M. McDonald M. (1999). Car-following: a historical review. Transport. Res. F Traffic Psychol. Behav. 2, 181–196. 10.1016/S1369-8478(00)00005-X

[B3] Chen Y. (2022). Research on collaborative innovation of key common technologies in new energy vehicle industry based on digital twin technology. Energy Rep. 8, 15399–15407. 10.1016/j.egyr.2022.11.120

[B4] Du Y. Qin B. Zhao C. Zhu Y. Cao J. Ji Y. (2021). A novel spatio-temporal synchronization method of roadside asynchronous mmw radar-camera for sensor fusion. IEEE Trans. Intell. Transport. Syst. 23, 22278–22289. 10.1109/TITS.2021.3119079

[B5] FHWA (2008). The Next Generation Simulation (NGSIM) [*Online*]. Available online at: http://www.ngsim.fhwa.dot.gov/

[B6] Fu R. Chen J. Zeng S. Zhuang Y. Sudjianto A. (2019). Time series simulation by conditional generative adversarial net. arXiv preprint arXiv:1904.11419. 10.2139/ssrn.3373730

[B7] Gao H. Shi G. Xie G. Cheng B. (2018). Car-following method based on inverse reinforcement learning for autonomous vehicle decision-making. Int. J. Adv. Robot. Syst. 15, 1729881418817162. 10.1177/1729881418817162

[B8] Gao N. Xue H. Shao W. Zhao S. Qin K. K. Prabowo A.. (2022). Generative adversarial networks for spatio-temporal data: a survey. ACM Trans. Intell. Syst. Technol. 13, 1–25. 10.1145/3474838

[B9] Goodfellow I. Pouget-Abadie J. Mirza M. Xu B. Warde-Farley D. Ozair S.. (2020). Generative adversarial networks. Commun. ACM. 63, 139–144. 10.1145/3422622

[B10] Greveling D. P. (2018). Modelling human driving behaviour using Generative Adversarial Networks (Ph.D. thesis). Faculty of Science and Engineering.

[B11] Gu Z. Li Z. Di X. Shi R. (2020). An lstm-based autonomous driving model using a waymo open dataset. Appl. Sci. 10, 2046. 10.3390/app10062046

[B12] He Z. Zheng L. Guan W. (2015). A simple nonparametric car-following model driven by field data. Transport. R. B Methodol. 80, 185–201. 10.1016/j.trb.2015.07.010

[B13] Huang X. Sun J. Sun J. (2018). A car-following model considering asymmetric driving behavior based on long short-term memory neural networks. Transport. Res. C Emerg. Technol. 95, 346–362. 10.1016/j.trc.2018.07.022

[B14] Huang Z. Wu J. Lv C. (2021). Driving behavior modeling using naturalistic human driving data with inverse reinforcement learning. IEEE Trans. Intell. Transport. Syst. 23, 10239–10251. 10.1109/TITS.2021.3088935

[B15] Kingma D. P. Ba J. (2014). Adam: a method for stochastic optimization. arXiv preprint arXiv:1412.6980. 10.48550/arXiv.1412.6980

[B16] Kuefler A. Morton J. Wheeler T. Kochenderfer M. (2017). “Imitating driver behavior with generative adversarial networks,” in 2017 IEEE Intelligent Vehicles Symposium (IV) (Los Angeles, CA: IEEE), 204–211.

[B17] Li J. Monroe W. Shi T. Jean S. Ritter A. Jurafsky D. (2017). Adversarial learning for neural dialogue generation. arXiv preprint arXiv:1701.06547. 10.18653/v1/D17-1230

[B18] Li L. Jiang R. He Z. Chen X. M. Zhou X. (2020). Trajectory data-based traffic flow studies: a revisit. Transport. Res. C Emerg. Technol. 114, 225–240. 10.1016/j.trc.2020.02.016

[B19] Li P. Y. Shrivastava A. (2002). Traffic flow stability induced by constant time headway policy for adaptive cruise control vehicles. Transport. Res. C Emerg. Technol. 10, 275–301. 10.1016/S0968-090X(02)00004-9

[B20] Lin L. Li W. Bi H. Qin L. (2021). Vehicle trajectory prediction using lstms with spatial-temporal attention mechanisms. IEEE Intell. Transport. Syst. Mag. 14, 197–208. 10.1109/MITS.2021.3049404

[B21] Liu Z. Wang Y. Feng J. (2022). Vehicle-type strategies for manufacturer's car sharing. Kybernetes 2022, 1095. 10.1108/K-11-2021-1095

[B22] Ma L. Qu S. (2020). A sequence to sequence learning based car-following model for multi-step predictions considering reaction delay. Transport. Res. C Emerg. Technol. 120, 102785. 10.1016/j.trc.2020.102785

[B23] Ma L. Qu S. Ren J. Zhang X. (2023). Mixed traffic flow of human-driven vehicles and connected autonomous vehicles: String stability and fundamental diagram. Math. Biosci. Eng. 20, 2280–2295. 10.3934/mbe.2023107 36899534

[B24] Mirza M. Osindero S. (2014). Conditional generative adversarial nets. arXiv preprint arXiv:1411.1784. 10.48550/arXiv.1411.1784

[B25] Mitchell M. (1998). An Introduction to Genetic Algorithms. Cambridge, MA: MIT Press.

[B26] Mo Z. Di X. (2022). “Uncertainty quantification of car-following behaviors: physics-informed generative adversarial networks,” in The 28th ACM SIGKDD in Conjunction With the 11th International Workshop on Urban Computing (UrbComp2022).

[B27] Montanino M. Punzo V. (2013). Reconstructed NGSIM I80-1. COST ACTION TU0903-MULTITUDE. Available online at: http://www.multitude-project.eu/exchange/101.html

[B28] Montanino M. Punzo V. (2015). Trajectory data reconstruction and simulation-based validation against macroscopic traffic patterns. Transport. Res. B Methodol. 80, 82–106. 10.1016/j.trb.2015.06.010

[B29] Papathanasopoulou V. Antoniou C. (2015). Towards data-driven car-following models. Transport. Res. C Emerg. Technol. 55, 496–509. 10.1016/j.trc.2015.02.016

[B30] Sachdeva N. Wang Z. Han K. Gupta R. McAuley J. (2022). “Gapformer: Fast autoregressive transformers meet rnns for personalized adaptive cruise control,” in 2022 IEEE 25th International Conference on Intelligent Transportation Systems (ITSC) (Macau: IEEE), 2528–2535.

[B31] SAE (2021). Taxonomy and Definitions for Terms Related to Driving Automation Systems for On-Road Motor Vehicles J3016_202104. Available online at: https://www.sae.org/standards/content/j3016_202104

[B32] Saifuzzaman M. Zheng Z. (2014). Incorporating human-factors in car-following models: a review of recent developments and research needs. Transport. Res. C Emerg. Technol. 48, 379–403. 10.1016/j.trc.2014.09.008

[B33] Shi H. Dong S. Wu Y. Li S. Zhou Y. Ran B. (2022). Generative adversarial network for car following trajectory generation and anomaly detection. Available at SSRN 4111253. 10.2139/ssrn.4111253

[B34] Shi K. Wu Y. Shi H. Zhou Y. Ran B. (2022). An integrated car-following and lane changing vehicle trajectory prediction algorithm based on a deep neural network. Physica A 599, 127303. 10.1016/j.physa.2022.127303

[B35] Toledo T. Koutsopoulos H. N. Ahmed K. I. (2007). Estimation of vehicle trajectories with locally weighted regression. Transpot. Res. Rec. 1999, 161–169. 10.3141/1999-17

[B36] Treiber M. Hennecke A. Helbing D. (2000). Congested traffic states in empirical observations and microscopic simulations. Phys. Rev. E 62, 1805. 10.1103/PhysRevE.62.1805 11088643

[B37] Treiber M. Kesting A. Helbing D. (2006). Delays, inaccuracies and anticipation in microscopic traffic models. Physica A 360, 71–88. 10.1016/j.physa.2005.05.001

[B38] Wang X. Jiang R. Li L. Lin Y. Zheng X. Wang F.-Y. (2017). Capturing car-following behaviors by deep learning. IEEE Trans. Intell. Transport. Syst. 19, 910–920. 10.1109/TITS.2017.2706963

[B39] Wang X. Jiang R. Li L. Lin Y.-L. Wang F.-Y. (2019). Long memory is important: a test study on deep-learning based car-following model. Physica A 514, 786–795. 10.1016/j.physa.2018.09.136

[B40] Wei D. Liu H. (2013). Analysis of asymmetric driving behavior using a self-learning approach. Transport. Res. B Methodol. 47, 1–14. 10.1016/j.trb.2012.09.003

[B41] Wu C. Kreidieh A. Parvate K. Vinitsky E. Bayen A. M. (2017). Flow: architecture and benchmarking for reinforcement learning in traffic control. arXiv preprint arXiv:1710.05465,05410. 10.48550/arXiv.1710.05465

[B42] Wu D. Luo X. Wang G. Shang M. Yuan Y. Yan H. (2017). A highly accurate framework for self-labeled semisupervised classification in industrial applications. IEEE Trans. Ind. Inform. 14, 909–920. 10.1109/TII.2017.2737827

[B43] Xu J. Pan S. Sun P. Z. Park S. H. Guo K. (2022). Human-factors-in-driving-loop: driver identification and verification via a deep learning approach using psychological behavioral data. IEEE Trans. Intell. Transport. Syst. 24, 3383–3394. 10.1109/TITS.2022.3225782

[B44] Xu J. Park S. H. Zhang X. Hu J. (2021). The improvement of road driving safety guided by visual inattentional blindness. IEEE Trans. Intell. Transport. Syst. 23, 4972–4981. 10.1109/TITS.2020.3044927

[B45] Yan H. He J. Zhao Y. Zhang L. Zhu C. Wu D. (2020). Gentiana macrophylla response to climate change and vulnerability evaluation in china. Global Ecol. Conservat. 22, e00948. 10.1016/j.gecco.2020.e00948

[B46] Yang L.-H. Zhang C. Chou X.-Y. Li S. Wang H. (2019). Research progress on car-following models. J. Traffic Transport. Eng. 19, 125–138. 10.19818/j.cnki.1671-1637.2019.05.013

[B47] Zhou M. Qu X. Li X. (2017). A recurrent neural network based microscopic car following model to predict traffic oscillation. Transport. Res. C Emerg. Technol. 84, 245–264. 10.1016/j.trc.2017.08.027

[B48] Zhou Y. Fu R. Wang C. Zhang R. (2020). Modeling car-following behaviors and driving styles with generative adversarial imitation learning. Sensors 20, 5034. 10.3390/s20185034 32899773 PMC7571238

[B49] Zhu M. Du S. S. Wang X. Pu Z. Wang Y.. (2022). Transfollower: Long-sequence car-following trajectory prediction through transformer. arXiv preprint arXiv:2202.03183. 10.2139/ssrn.4086626

[B50] Zhu M. Wang X. Wang Y. (2018). Human-like autonomous car-following model with deep reinforcement learning. Transport. Res. C Emerg. Technol. 97, 348–368. 10.1016/j.trc.2018.10.024

